# Docosahexaenoic Acid Protects Muscle Cells from Palmitate-Induced Atrophy

**DOI:** 10.5402/2012/647348

**Published:** 2012-09-30

**Authors:** Randall W. Bryner, Myra E. Woodworth-Hobbs, David L. Williamson, Stephen E. Alway

**Affiliations:** Division of Exercise Physiology, School of Medicine, West Virginia University, Morgantown, WV 26506-9227, USA

## Abstract

*Background*. Accumulation of free fatty acids leads to lipid-toxicity-associated skeletal muscle atrophy. Palmitate treatment reduces myoblast and myotube growth and causes apoptosis *in vitro*. It is not known if omega-3 fatty acids will protect muscle cells against palmitate toxicity. Therefore, we examined the effects of docosahexaenoic acid (DHA) on skeletal muscle growth. *Methods*. Mouse myoblasts (C_2_C_12_) were differentiated to myotubes, and then treated with 0 or 0.5 mM palmitic acid or 0 or 0.1 mM DHA. *Results*. Intramyocellular lipid was increased in palmitate-treated cells but was prevented by DHA-palmitate cotreatment. Total AMPK increased in DHA+ palmitate-treated compared to palmitate only cells. RpS6 phosphorylation decreased after palmitate (−55%) and this was blunted by DHA+ palmitate (−35%) treatment. Palmitate treatment decreased PGC1*α* protein expression by 69%, but was increased 165% with DHA+ palmitate (*P* = 0.017) versus palmitate alone. While palmitate induced 25% and 90% atrophy in myotubes (after 48 hours and 96 hours, resp.), DHA+ palmitate treatment caused myotube hypertrophy of ~50% and 100% after 48 and 96 hours, respectively. *Conclusion*. These data show that DHA is protective against palmitate-induced atrophy. Although DHA did not activate the AMPK pathway, DHA treatment restored growth-signaling (i.e., rpS6) and rescued palmitate-induced muscle atrophy.

## 1. Background

Skeletal muscle mass and structure are negatively affected by obesity and excessive uptake of fatty acids. In cardiac muscle, this is thought to be due, at least in part, to a lipid-induced increase in oxidative stress and dysfunction [1], although the role in skeletal muscle has been less well studied. It is known that obese individuals have fewer type I muscle fibers and more type IIb fibers compared with lean individuals [2]. Similarly, obese leptin deficient rats have less muscle mass than lean rodents of the same age [3], and genetically obese ob/ob mice have reduced muscle mass and the ability to undergo hypertrophy compared with lean litter mates [4]. In addition, aging appears to accelerate muscle loss in obese persons [5] and a primary cause for this could be a reduced rate of muscle protein synthesis in response to hormonal and nutrition stimuli. A loss of muscle would significantly reduce the amount of metabolically active tissue available to oxidize fatty acids and could therefore augment those mechanisms associated with insulin resistance and diabetes in a chronically overnourished state. Understanding the link between lipid metabolism and muscle loss is of clinical significance for obesity, type 2 diabetes, and sarcopenia.

The composition of intracellular fatty acid [6–8] may determine if it is detrimental to muscle mass or function. For example, saturated fatty acids lead to insulin resistance [6, 9], while exposure to unsaturated fatty acids prevents [6, 7, 9–11], attenuates [12], or reverses [13–15] insulin resistance. Unsaturated fatty acids may rescue the detrimental metabolic effects associated with saturated fatty acid treatment alone [9]. Of particular interest are the long chain omega-3 (n-3) polyunsaturated FA (PUFA), such as docosahexaenoic acid (DHA) which attenuate the reductions in insulin sensitivity caused by excessive lipids [7, 8, 10] and sucrose [13–15] in cells [7], metabolically normal [10, 11, 16, 17], obese [12], and insulin-resistant animals [13–15]. Omega PUFAs partition fatty acids toward oxidation [18], which may provide a mechanism to improve insulin sensitivity by promoting skeletal muscle fatty acid oxidation and reducing intramyocellular lipid content.

Palmitate is the most abundant systemic saturated fatty acid, and therefore it has received considerable attention in investigations on dyslipidemia on various tissues [19–23]. Treatment of different cell types with palmitate *in vitro *has resulted in apoptosis [3, 21, 24, 25] and has been found to inhibit Akt/Protein kinase B (Akt) activity in response to insulin [19, 26, 27]. These observations are consistent with muscle atrophy in models of insulin resistance [3].

Treatments that activate AMP-activated protein kinase (AMPK), have been shown to improve insulin sensitivity [28–34]. Furthermore, the ability of AMPK to enhance fatty acid oxidation and improve oxidative capacity could reduce intramyocellular lipid content. Long-term fatty acid treatments have been demonstrated to inhibit AMPK [29, 35] and reduce insulin sensitivity [29] in rodent skeletal muscle, while n-3 PUFAs are shown to attenuate reductions in insulin sensitivity associated with nutrient oversupply [8, 14, 36, 37]. However, it is not known if n-3 PUFAs directly affect AMPK signaling in muscle cells. Therefore, the purpose of this study was to determine if n-3 PUFA treatment is protective against palmitate-associated muscle cell atrophy, by enhancing AMPK signaling and reducing intramyocellular lipid content.

## 2. Methods

### 2.1. Materials

Mouse C_2_C_12_ myoblasts were purchased from American Type Culture Collection (ATCC, Manassas, VA). Fetal calf serum (FCS) was purchased from Atlanta Biologicals (Lawrenceville, GA). ITS Liquid Media Supplement, palmitic acid sodium salt, cis-4, 7, 10, 13, 16, 19-docosahexaenoic acid (DHA) oil, insulin, Ponceau S red, 99% triethyl phosphate, and a citrate synthase assay kit (Cat# CS0720) were purchased from (Sigma-Aldrich, St. Louis, MO). BSA was purchased from Santa Cruz Biotechnology (Santa Cruz, CA). SDS-PAGE precast gels were purchased from Invitrogen (Carlsbad, CA), and nitrocellulose membranes and an *RC DC* protein assay kit (500-0121) were purchased from Bio-Rad (Hercules, CA). Antibodies were purchased from Cell Signaling Technology (Cell Signaling Technology, MA, USA), and goat anti-rabbit and goat anti-mouse horseradish peroxidase-conjugated IgG were purchased from Jackson ImmunoResearch Laboratories, Inc. (West Grove, PA). The chemiluminescence substrate for developing the GAPDH protein blots was Pierce Enhanced Chemiluminescence (Thermo Fisher Scientific Rockford, IL), and for all other proteins, was Advanced Enhanced Chemiluminescence (GE Healthcare, Piscataway, NJ). ReBlot Plus Strong Solution was purchased from Millipore (Billerica, MA). X-ray film was purchased from Eastman Kodak (Rochester, NY, USA). Oil Red O powder was purchased from Fluka Analytical (Fluka Analytical/Sigma-Aldrich, St. Louis, MO).

### 2.2. Cell Culture

Mouse C_2_C_12_ myoblasts (American Type Culture Collection, Manassas, VA) were seeded in six-well (35 mm) plates in Dulbecco Modified Eagle's Medium (DMEM, Invitrogen, Carlsbad, CA) supplemented with 10% fetal calf serum (Atlanta Biologicals, Lawrenceville, GA) and 1% penicillin and streptomycin (Invitrogen, Carlsbad, CA) and maintained in a humidified incubator at 37°C in an atmosphere of 5% CO_2_. Cells were grown to ~95% confluence and then induced to differentiate into myotubes by incubation in serum- and PS-free DMEM supplemented with 1% ITS Liquid Media Supplement (Sigma-Aldrich, St. Louis, MO) for 3 days. After differentiation, cells were maintained in DMEM with 2% fetal calf serum until experimental treatment. All experiments were performed in triplicate, with each experiment repeated three times for a total of 9 samples for each data point. Each mean was calculated from 3 independent experiments.

Palmitate and cis-4, 7, 10, 13, 16, 19-docosahexaenoic acid (DHA) oil (Sigma-Aldrich,St. Louis, MO) was administered to cells as described by Chavez and Summers [19, 26]. Briefly, DHA was dissolved in ethanol and diluted in DMEM containing 2% BSA to reach desired fatty acid concentrations. For dose-response experiments, myotubes were treated separately with 0 mM, 0.1 mM, 0.25 mM, 0.5 mM, 0.75 mM, and 1.0 mM concentrations of palmitate and DHA, containing 2% FCS, and 2% BSA for 24 hours. For time-response experiments, myotubes were treated with media containing 2% FCS 2% BSA, and 0.5 mM palmitate or 0.1 mM DHA for 24, 48, and 96 hours. For all subsequent experiments, myotubes were treated with media containing 2% FCS, 2% BSA, and 0.5 mM palmitate, 0.1 mM DHA, 0.5 mM palmitate plus 0.1 mM DHA, or no fatty acids for 96 hours, and fresh media was provided every 48 hours. For insulin-stimulation experiments, myotubes were washed once with PBS and treated with 100 nM insulin in DMEM for 15 minutes. An additional vehicle-only control group (containing DMEM, BSA, and ethanol) was also included in all control experiments.

### 2.3. Cell Morphology

Images from myotubes that were treated for 48 or 96 hours were visualized at ×20 magnification using an inverted light microscope (Olympus America Inc., Melville, NY) with a digital camera and captured with a Spot RT camera and Spot Software (Diagnostic Instruments, Sterling Heights, MI). Myotube diameter was measured from randomly selected microscope fields from three different wells of control and treated cells (12 wells total per time-point) using Image J software (37). Six diameters were measured per myotube, and ten myotubes were measured per well, except in the case of palmitate-treated cells, where if ten myotubes were not present, all of the remaining myotubes were measured.

### 2.4. Evaluation of Phosphorylated and Total Proteins

The myotubes were harvested in sodium dodecyl sulfate (SDS) sample buffer (1% SDS, 6 mg/mL EDTA, 0.06 M Tris (hydroxymethyl) aminomethane (pH 6.8), 2 mg/mL bromophenol blue, 15% glycerol, and 5%  *β*-mercaptoethanol). Protein concentrations were quantified in duplicate using an *RC DC* protein assay (500-012; BioRad, Hercules, CA) and averaged for determination of Western blot loading volumes. The proteins from myoblast samples were separated by electrophoresis using 10%, 3–8%, or 4–12% SDS-PAGE gels (Invitrogen, Carlsbad, CA). Control and treated lysates were loaded on the same gel to account for possible variations between blots. A standard molecular weight marker was added to one lane to verify protein sizes in each gel. The proteins were transferred to a nitrocellulose membrane and stained with Ponceau S red (Sigma-Aldrich, St. Louis, MO) to confirm a uniform transfer of proteins to the membrane. The membranes were probed with primary antibodies against phosphorylated T172 for AMPK*α*, S79 for ACC, serine 636/639 for IRS-1, serine 473 for Akt, S21/9 for GSK3*α*/*β*, S240/244 for rpS6, or for total protein expression of PGC1*α* and COX-IV (Cell Signaling Technology, MA, USA). Membranes were incubated with the appropriate conjugated horseradish peroxidase secondary antibodies (Jackson ImmunoResearch Laboratories, Inc., West Grove, PA). The protein bands were visualized by exposing the membranes to X-ray film (BioMax MS-1, Eastman Kodak, Rochester, NY, USA). The membranes were stripped with 1X ReBlot Plus Strong Stripping Solution (Millpore, Billerica, MA) and probed with antibodies against total protein expression of AMPK*α*, ACC, Akt, GSK3*β*, *β*-tubulin, and GAPDH (Cell Signaling Technology, MA, USA). The signals for GAPDH were developed by Pierce Enhanced Chemiluminescence (Thermo Fisher Scientific Rockford, IL) and for all other proteins by Advanced Enhanced Chemiluminescence (GE Healthcare, Piscataway, NJ). Digital records of the films were captured with a Kodak 290 camera, and bands were quantified as optical density × band area by a one-dimensional image analysis system (Eastman Kodak, Rochester, NY) and expressed in arbitrary units normalized to GAPDH.

### 2.5. Oil Red O Stain

Oil Red O (Fluka Analytical/Sigma-Aldrich, St. Louis, MO) staining was performed to determine the intramyocellular lipid content of myotubes after 48 and 96 hours [38]. The cells were grown, differentiated, and treated with fatty acids as described above. A stock solution of Oil Red O (5 g/L) was prepared in a 3 : 2 ratio of 99% triethyl phosphate to distilled water. For staining, the stock solution was diluted to a 36%, and filtered three times by passing through a syringe with a 0.45 micron filter. The plates were washed three times with PBS and the myotubes were fixed with 10% formalin then washed with distilled water and stained with the working solution of Oil Red O/triethyl phosphate. Stained myotubes were rinsed with distilled water and visualized by microcoscopy (Olympus America Inc., Melville, NY). Intramyocellular lipid content was quantified by measuring fluorescence (excitation 485 nm, emission 530 nm) of the stained lipids, and values were normalized to protein content per well, which was determined using a* RC DC* protein assay kit (500-0121; BioRad, Hercules, CA). 

### 2.6. Citrate Synthase Activity

To evaluate the effects of fatty acid treatments on mitochondria oxidative metabolism after 96 hours, citrate synthase (CS) activity was determined spectrophotometrically according to the method of Srere [39] slight modifications from that previously reported by [40]. Briefly, the myotubes were lysed in 200 *μ*L of CelLytic M Reagent (Sigma-Aldrich), centrifuged at 12,000 ×g, and the supernatant transferred to a chilled test tube. Protein content was determined as described previously. The assay consisted of 100 mM Tris buffer (pH 8.35), 5 mM 5,5-dithiobis(2-nitrobenzoate) (DTNB), 22.5 mM acetyl-CoA, 25 mM oxaloacetate (OAA), and 10 *μ*L of sample lysate in a 96-well plate. The color change was monitored at wavelength of 405 nm at 15-s intervals for a period of 3 min by using a Synergy HT Multi-Mode microplate reader (Bio Tek, Winooski, VT). All samples were evaluated in triplicate. CS activity for each sample was normalized to protein content. A CS positive control was included for each experiment.

### 2.7. Statistical Analyses

The data are presented as the mean percent change ± standard error for a minimum of three cell culture experiments (*n* = 3) in triplicate. The insulin-stimulation experiments consisted of two experiments (*n* = 2), each completed in triplicate. A One-Way Analysis of Variance with Tukey post hoc analysis was used to evaluate differences for each variable between treatments, and statistical significance was set at *P* ≤ 0.05. Analyses were conducted using SPSS 12.0.1 software package.

## 3. Results

### 3.1. Selection of Fatty Acid Doses and Treatment Duration. 

**Figure 1:**
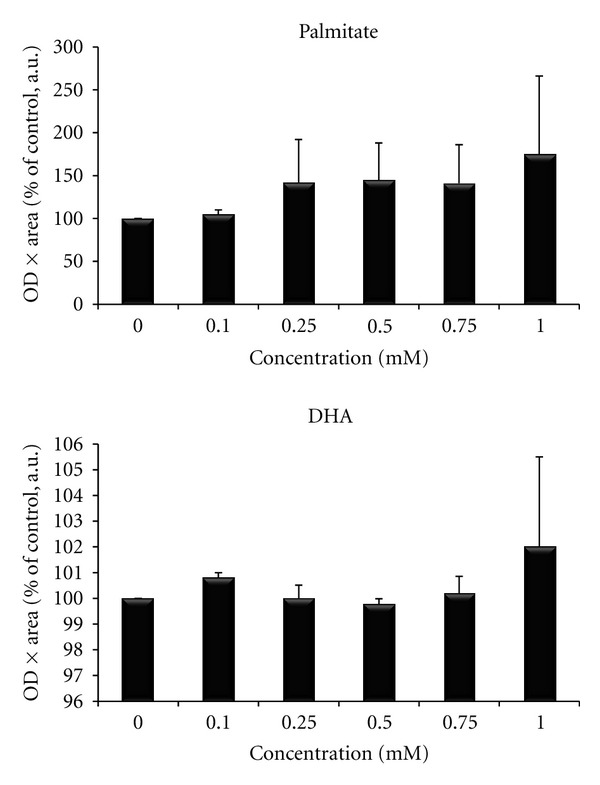
Dose-response curves for activation of AMPK following palmitate and cis-4, 7, 10, 13, 16, 19-docosahexaenoic acid (DHA) treatments.

Dose- and time-response curves were generated to evaluate both myotube morphology and levels of phosphorylated and total AMPK, since it was the primary protein of interest. A concentration of 0.5 mM palmitate and 0.1 mM DHA was chosen because it provided the greatest phosphorylation of AMPK without loss of cellular integrity (Figure 1). Furthermore, these concentrations gave a polyunsaturated: saturated fatty acid ratio of 0.2, which is similar to previous studies using a ratio of 0.25 [41].

**Figure 2:**
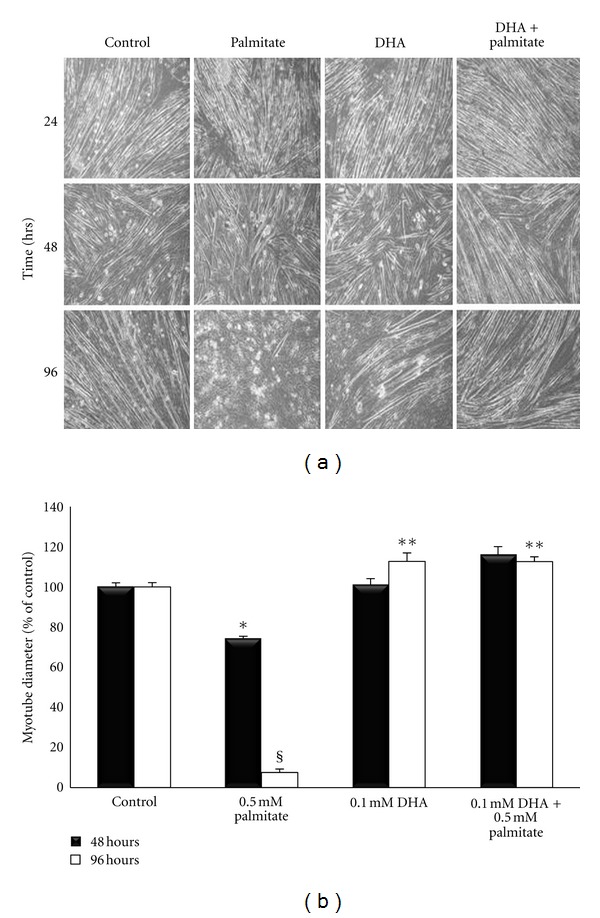
(a) Myotube morphology at 24, 48, and 96 hours of incubation. (b) Myotube diameter of treated cells. Six diameters per myotube from ~10 myotubes (per culture) from three wells per treatment condition that were treated for 48 or 96 hours with palmitate, DHA, DHA+palmitate, or no fatty acids. *Denotes *P* ≤ 0.05 versus other 48 h treatment conditions; ^§^Denotes *P* < 0.00 versus other 96 h treatment conditions; **Denotes *P* < 0.05 versus 96 h control conditions.

There were no apparent changes in the myotube morphology after 24 hours of treatment (Figure 2(a)), and therefore we used the 48 and 96 hour time-points for measurement of myotube diameter (Figure 2(b)). Palmitate treatment decreased myotube diameter by 25% (*P* = 0.052) after 48 hours and over 90% (*P* < 0.001) after 96 hours versus control. However, DHA maintained myotube morphology and diameter similar to that found after control treatment. Adding DHA to the palmitate treatment, increased myotube diameter by ~50% (*P* = 0.004) after 48 hours and over 100% (*P* < 0.001) after 96 hours versus palmitate alone.

### 3.2. Palmitate Treatment Increases Intramyocellular Lipid Content of Myotubes

**Figure 3:**
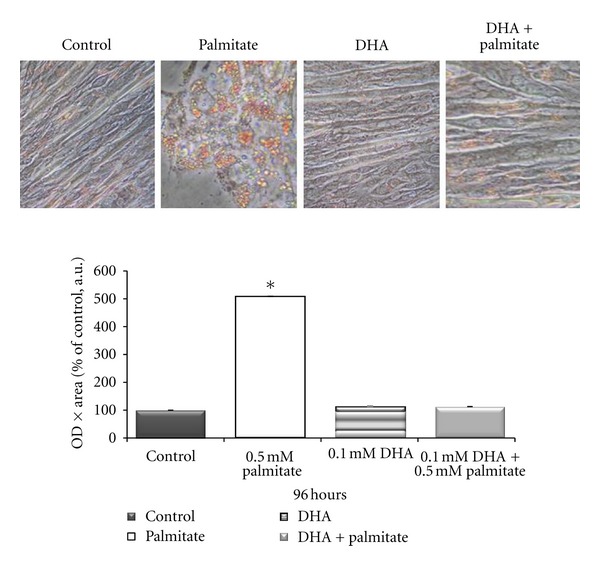
Lipid content of the cells incubated with either no fatty acids (Control), 0.5 mM palmitate, 0.1 mM cis-4, 7, 10, 13, 16, 19-docosahexaenoic acid (DHA), or 0.1mM DHA plus 0.5 mM palmitate for 96 hours and then treated with a 36% Oil red O/triethyl phosphate solution. Fluorescence was measured (excitation 485 nm, emission 530 nm) and normalized to the average protein content per treatment and expressed as arbitrary units (a.u.). *Denotes *P* < 0.0001 versus all other conditions.

Because the most dramatic change in myotube morphology and size without complete loss of palmitate cells occurred at 96 hours, we chose this time-point to conduct subsequent measurements. Since AMPK is considered to be a regulator of lipid homeostasis in skeletal muscle, the effect of different fatty acid treatments on intramyocellular lipid content was evaluated. Myotubes were stained with Oil red O, which indicates the levels of all neutral lipids. There was a 400% (*P* < 0.000) increase in auto fluorescence of Oil red O stained myotubes with palmitate treatment, when normalized to the average protein content per treatment. Intramyocellular lipid content was not different from control levels in DHA-palmitate cotreated myotube cultures (Figure 3).

**Figure 4:**
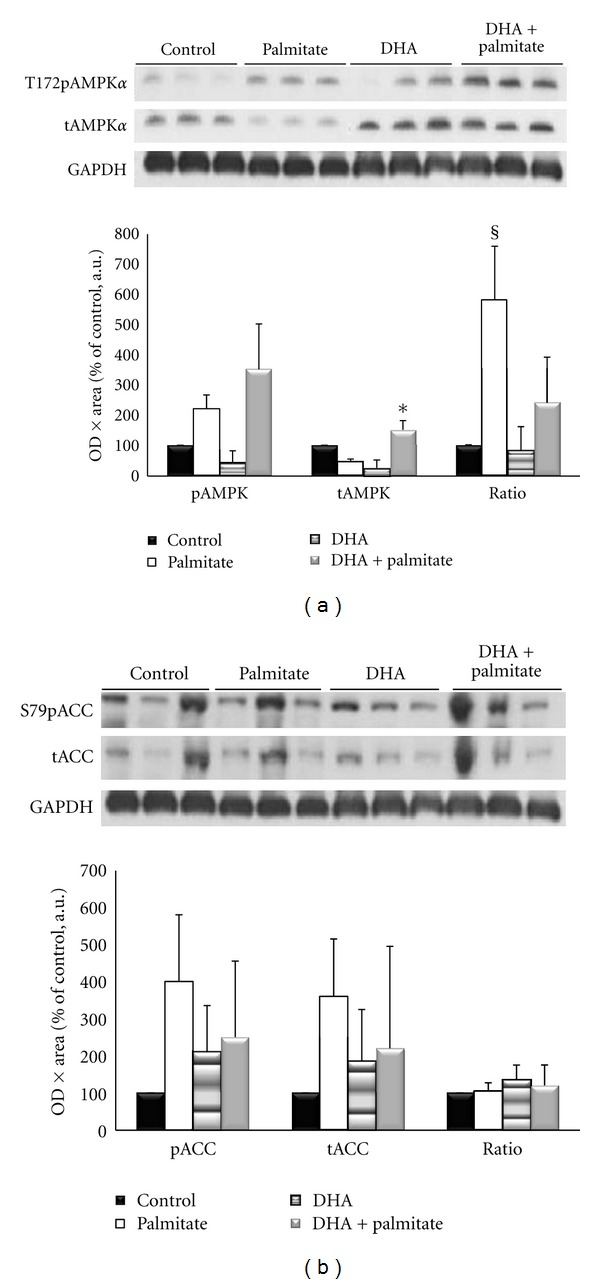
(a) The phosphorylated AMPK*α* (p-AMPK), total AMPK*α* (t-AMPK), and ratio of p-AMPK*α*/t-AMPK*α* after 96 hours of incubation. (b) The phosphorylated ACC (p-ACC), total ACC (t-ACC), and ratio of p-ACC/t-ACC after 96 hours of incubation. *Denotes *P* ≤ 0.05 versus palmitate conditions for total AMPK*α*. ^§^Denotes *P* < 0.05 versus all other conditions for the AMPK ratio.

To determine if the maintenance of myotube morphology and intracellular lipid content with DHA treatment was due to activation of the AMPK pathway, we measured phosphorylation of AMPK*α* on Thr172, which is required for its activation [42], and total AMPK protein expression (Figure 4(a)). Phospho-AMPK*α* ^Thr172^ levels were not significantly different between treatments, but addition of DHA to the palmitate treatment led to 106% higher (*P* = 0.05) total AMPK levels than palmitate alone. This was associated with a 5.7-fold increase (*P* = 0.032) in the AMPK ratio in palmitate as compared to control conditions. To determine if the activation of AMPK with palmitate treatment was propagated downstream, we examined its cytosolic target, acetyl Co-A carboxylase (ACC) (Figure 4(b)). While all fatty acid treatments led to increases in phospho-ACC^Ser79^ levels, there were no significant differences between treatments or control conditions. These data are consistent with the phospho-AMPK*α* ^Thr172^ data (Figure 4(a)). The total ACC levels mirrored its phosphorylated levels and were also not significantly different between treatments; therefore, the ACC ratio was also similar between treatments (Figure 4(b)).

### 3.3. DHA Maintains Protein Abundance of Oxidative Markers in Palmitate-Treated Myotubes

**Figure 5:**
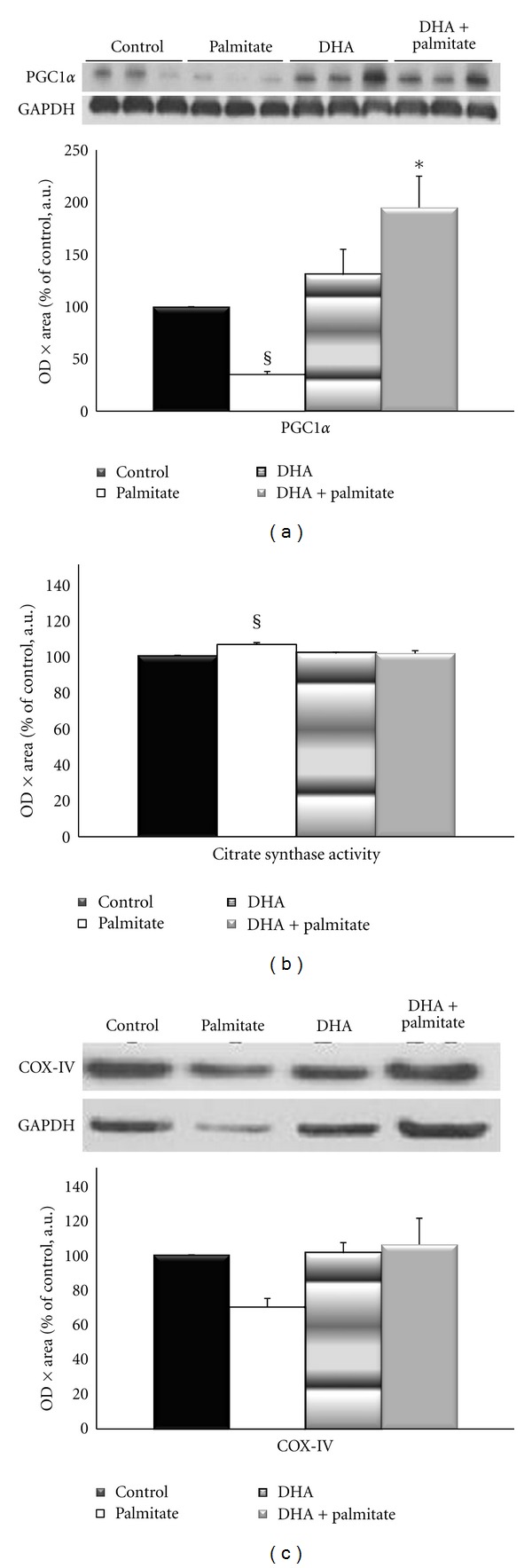
(a) Protein expression of peroxisome proliferator-activated receptor gamma coactivator 1*α* (PGC1*α*) following 96 hours of incubation. (b) Citrate synthase (CS) activity of cells following 96 hours of incubation. (c) Cytochrome c oxidase subunit IV (COX-IV) protein expression following. *Denotes *P* < 0.05 versus palmitate condition. ^§^Denotes *P* < 0.05 versus all other conditions.

Since AMPK is also known to activate transcription for long-term regulation of lipid homeostasis [43], the total protein expression of its nuclear target, PGC1*α*, was measured. Palmitate treatment decreased PGC1*α* protein expression by 69% versus control, whereas the addition of DHA to the palmitate treatment completely attenuated this effect by increasing its protein expression 165% (*P* = 0.017) versus palmitate treatment alone (Figure 5(a)). This suggests that DHA preserves oxidative metabolic capacity in palmitate-treated cells. To determine if the improvement in PGC1*α* expression with DHA was matched downstream by an increase in oxidative metabolism, we measured citrate synthase activity as a marker of the tricarboxylic acid cycle and COX-IV protein expression as an indicator of the of the electron transport chain. We found disparate effects on these oxidative markers. Citrate synthase activity demonstrated a small but significant 3% increase (*P* < 0.05) with palmitate treatment versus all other conditions, and addition of DHA to palmitate had similar CS activity as control cells (Figure 5(b)). However, palmitate treatment led to a 34% decrease (*P* = 0.297) in COX-IV protein expression, while addition of DHA returned COX-IV expression to control levels (Figure 5(c)).

### 3.4. DHA Attenuates Palmitate-Induced Detriments in the Insulin Signaling Pathway

**Figure 6:**
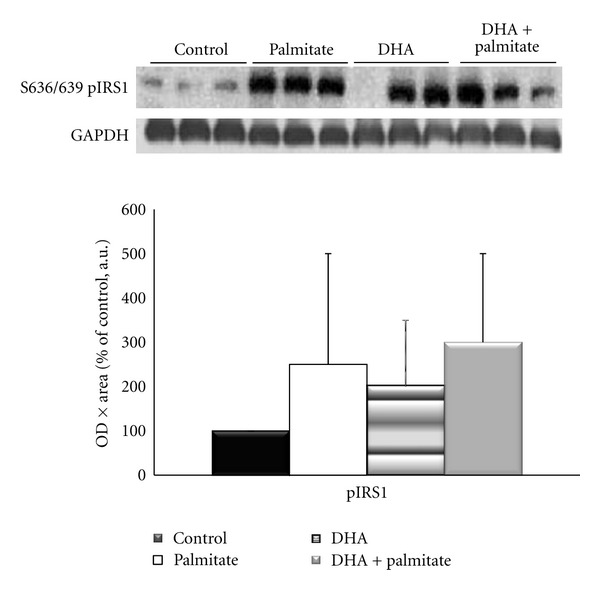
Protein expression of IRS-1 phosphorylation on S636/639 and normalized to glyceraldehyde-3-phosphate dehydrogenase (GAPDH) protein expression following 96 hours of incubation.

**Figure 7:**
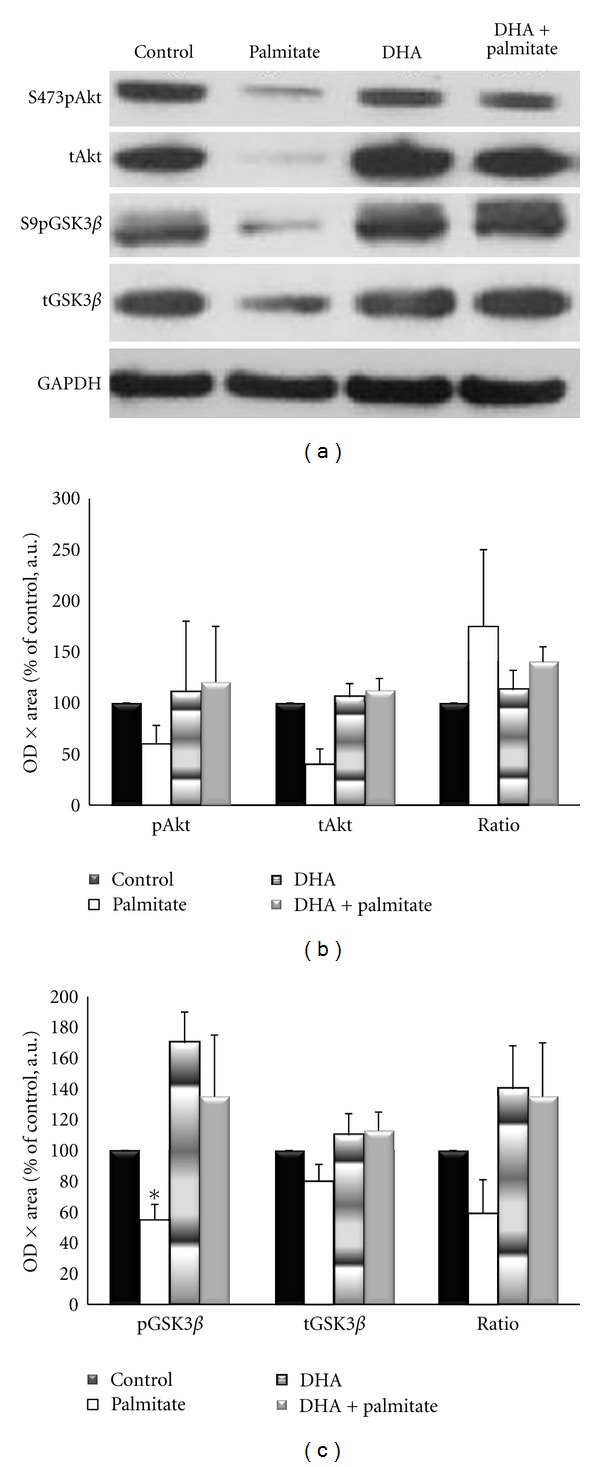
(a) Representative Western blots for phospho-Akt^Ser473^, phospho-GSK3*β* ^Ser9^, total Akt, and GSK3*β*. (b) Basal phosphorylated-Akt (p-Akt), total Akt (t-Akt), and the ration of p-Akt/t-Akt following 96 hours of incubation. (c) Basal phosphorylation of GSK3*β* ^Ser9^ (p-GSK3*β*), total GSK3*β* (t-GSK3*β*), and p-GSK3*β*/ t-GSK3*β* following 96 hours of incubation.

**Figure 8:**
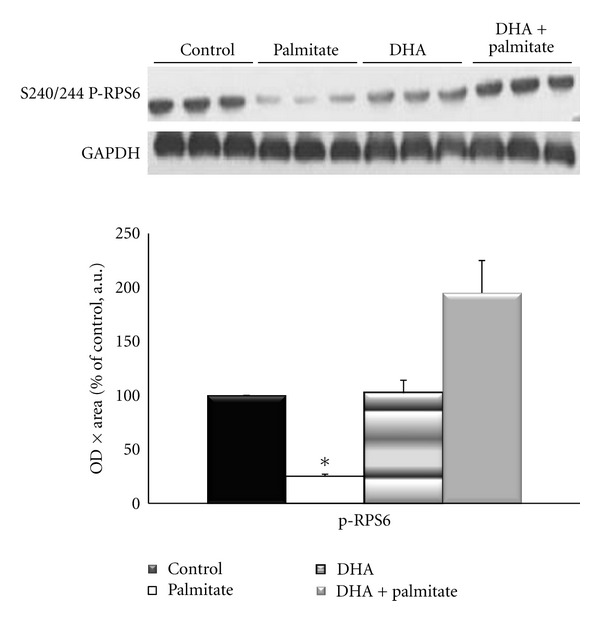
Protein expression of ribosomal protein S6 (rpS6) phosphorylation on Ser240/244 and normalized to glyceraldehyde-3-phosphate dehydrogenase (GAPDH) protein expression following 96 hours of incubation. *Denotes *P* < 0.05 versus palmitate condition.

To determine if changes in intramyocellular lipid content and markers of oxidative metabolism with DHA treatment led to alterations in the insulin signaling pathway, the inhibitory serine phosphorylation site of the insulin receptor substrate (IRS) 1 and the downstream proteins Akt, GSK3*β*, and rpS6 were examined. All fatty acid treatments elevated p-IRS-1^Ser636/639^ by 2-3-fold, although these increases were not significant from each other or control conditions (Figure 6). The phosphorylation of Akt on Ser473 was measured because it is required for its activation [44], and previous research has demonstrated it to be decreased with palmitate treatment in skeletal muscle [45–47]. Although not statistically significant, Akt phosphorylation and total protein were decreased by ~33% and phospho-GSK3*β* by ~50% with palmitate treatment as compared to control conditions, while the addition of DHA completely attenuated these decreases (Figures 7(a) and 7(b)). However, contrary to the Akt data, total GSK3*β* levels remained unchanged (Figures 7(a) and 7(c)). Palmitate decreased phospho-rpS6^Ser240/244^ levels to ~25% of control, while the addition of DHA increased its activation by 7-fold (*P* = 0.017) (Figure 8).

**Figure 9:**
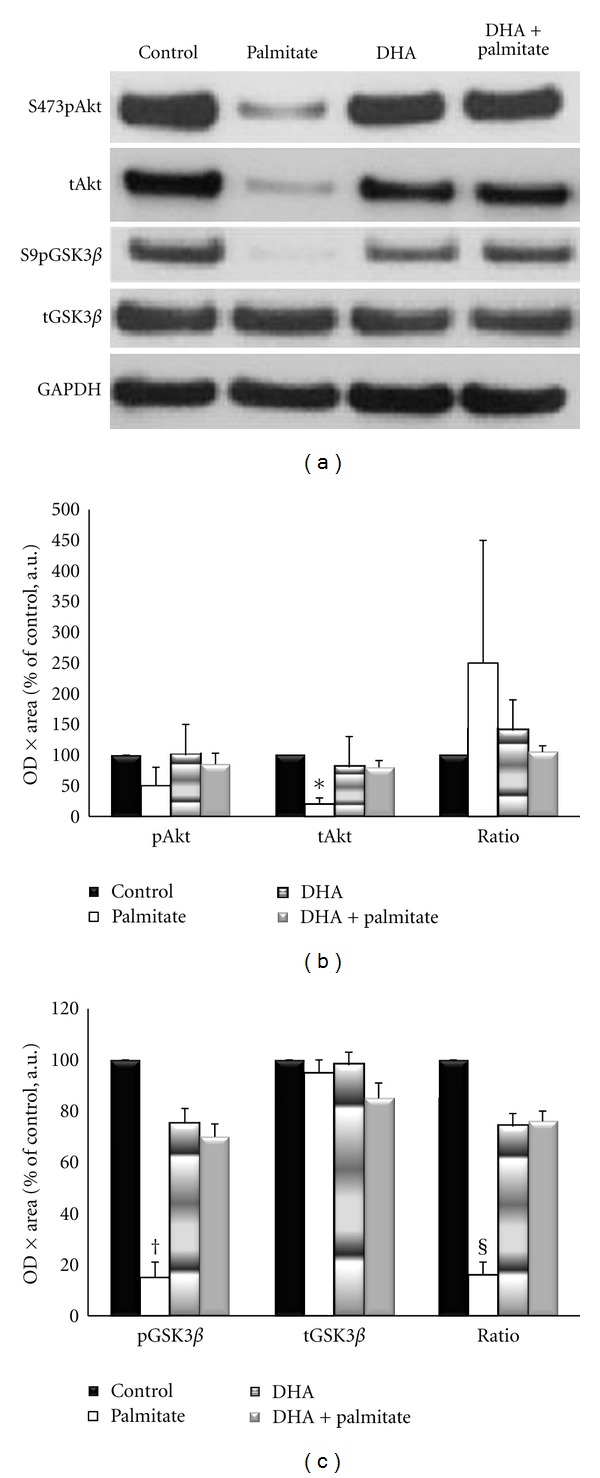
(a) Representative Western blots for phospho-Akt^Ser473^, phospho-GSK3*β* ^Ser9^, total Akt, and GSK3*β* with insulin stimulation. (b) Insulin-stimulated phosphorylated-Akt (p-Akt), total Akt (t-Akt), and the ratio of p-Akt/t-Akt following 96 hours of incubation. *Denotes *P* < 0.05 versus all other conditions. (c) Insulin-stimulated phosphorylation of GSK3*β* ^Ser9^ (p-GSK3*β*), total GSK3*β* (t-GSK3*β*), and p-GSK3*β*/t-GSK3*β* following 96 hours of incubation. ^†^Denotes *P* < 0.05 versus all other conditions for pGSK3*β*. ^§^Denotes *P* < 0.05 versus all other conditions for the GSK ratio.

To observe the responsiveness of the signaling pathway, myotubes were stimulated with 100 nM insulin for 15 minutes. This dose and treatment duration were chosen to elicit a maximal signaling response [48]. Overall, DHA attenuated the decrements of palmitate treatment (Figure 9). Phospho-Akt was reduced by ~50%, relative to palmitate treated cells, and total Akt protein expression was only ~25–45% of the other treatments (*P* < 0.02). Activation of GSK3*β* was also decreased 55–85% by palmitate (*P* < 0.03). Addition of DHA attenuated all of these decreases to approximately 70% of control values (*P* < 0.03). Together these data indicate a complete rescue of basal- and a partial but significant attenuation of insulin-stimulated signaling by adding the omega-3 polyunsaturated fatty acid DHA to the saturated fatty acid palmitate treatment.

## 4. Discussion

The novel findings of this study were that DHA was protective against the negative effects of palmitate on myotube size and morphology, specific measures of oxidative metabolism, intramyocellular lipid content, and insulin signaling, independent of AMPK activation. Overall these data are consistent with the general findings that omega-3 polyunsaturated fatty acids have the ability to prevent detrimental effects of saturated fatty acids [8, 10, 13, 41, 49, 50]. A most-striking initial finding of this research is that DHA not only prevented the myotube morphology and size loss with palmitate, but it also reversed the negative effects of palmitate on cell size. Indeed the anabolic effect of DHA was large because cotreatment of palmitate with DHA increased myotube diameter more than 12% over control cells after 4 days.

To determine if the changes in myotube morphology were associated with changes in protein expression and activation of signaling proteins involved in lipid metabolism, we measured AMPK phosphorylation and total AMPK protein expression. Contrary to our hypothesis, DHA did not appear to exert its positive effects through activation of AMPK since all fatty acid treatments led to a nonsignificant 2-3-fold increase in phosphorylated AMPK. However, there was a significant difference in the AMPK ratio between treatments, which was due to decreased total AMPK levels in palmitate-treated cells. The total AMPK data are consistent with findings that total AMPK*α* protein levels were decreased by approximately 60% after 5 months of high-fat feeding in rodents [29]. However, phospho-AMPK^Thr172^ levels have also been reported to decrease in high-fat feeding studies, [29], and this is contrary to our observations and could possibly reflect the differences between animal and cell culture models. The high AMPK ratio in the palmitate-treated cells indicates that most of the remaining total AMPK present in the cells was activated. The morphology of the cells treated with palmitate suggests that they maybe undergoing apoptosis and/or death (7) and were trying to produce energy by activating the master energetic regulator that stimulates ATP-producing processes [51]. Although we did not measure cell death in the current study, our lab has previous shown that 75 mM palmitate treatment of myotubes for 16 hours lead to a 7-fold increase in DNA fragmentation versus control-treated cells [46], and the activation of AMPK via AICAR treatment in differentiating C_2_C_12_ myoblasts led to increased DNA fragmentation and caspase-3 cleavage [52]. These data along with the morphological characteristics of the cells are consistent with the idea that the palmitate-treated cells in the current study were undergoing apoptosis. Conversely, DHA did not differentially increase AMPK phosphorylation, but it was able to maintain the AMPK ratio through blunting the decrease in total AMPK. It is possible that this contributed to the attenuation of cellular atrophy and death and improved myotubes morphology and size compared to palmitate treatment.

Activation of AMPK decreases expression of genes involved in lipid synthesis [53], and we had expected that palmitate treatment would have increased the phosphorylated : total AMPK ratio. Furthermore, increases in phosphorylation of AMPK, ACC, and lipids of 2.5- to-3-fold have been reported in L6 myotubes after palmitate treatment [54]. However, in our study, the cytosolic downstream target of AMPK, ACC did not have significant changes in ACC phosphorylation or total ACC levels. This leads to similar ratios of phosphorylated to total ACC in all conditions. However, intramyocellular lipid content was substantially increased in the palmitate-treated cells compared to the other conditions in the present study. Moreover, apart from measurements of AMPK activation, the conditions of obesity [55, 56] and high-fat feeding [11, 57] are shown to increase intramyocellular lipid content. Our data indicate that although DHA was able to reduce accumulation of intramyocellular lipids when added to the palmitate treatment, this alteration was not through activation of the AMPK pathway. These findings are consistent with data showing that *α*-lipoic acid improved insulin sensitivity and lowered lipid accumulation in mouse skeletal muscle without changes in phosphorylated AMPK or ACC [58]. One possibility is that activation of the AMPK pathway is not required for alterations in fatty acid-induced skeletal muscle lipid metabolism. On the other hand, it has been shown that myotubes treated with n-3 fatty acid eicosapentaenoic acid had low lipid accumulation, regulated a high number of genes involved in glucose utilization, and also increased IL-6 expression [59]. IL-6 is able to phosphorylate and activate AMPK in skeletal muscle and thereby regulate muscle substrate utilization [60]. We did not measure IL-6 in the current study, so we do not know if the failure to identify changes in AMPK signaling was related to a lack of change in IL-6 levels after treatment with DHA. Another possibility was that the reduced intramyocellular lipid content in the control and both DHA treatments of the current study was due to an increase in lipid oxidation versus palmitate conditions, resulting in lower net lipid content versus palmitate-treated cells. Nevertheless, the ACC data does not support this hypothesis. Although beyond the scope of this study, additional studies will be need to resolve these issues.

We hypothesized that the addition of DHA to the palmitate-treated myotubes would improve the cell's capacity for oxidative metabolism and thereby lower intramuscular lipid stores. Therefore we examined markers for oxidative enzymes and the transcription factor PGC1*α*. Coincubation of DHA to the palmitate treatment maintained PGC1*α* near control levels, which indeed suggests that DHA may prevent palmitate-induced decreases in oxidative metabolism and ultimately improve utilization of intramyocellular lipids. PGC1*α* has clearly been shown to be involved in the regulation of mitochondrial biogenesis [61], a process known to be important for the oxidation of lipids. The maintenance of PGC1*α* may have also attenuated the palmitate-induced cellular atrophy and/or death. Sandri et al. [62] demonstrated a sharp decrease in PGC1*α* mRNA expression in diabetes-induced atrophied muscle, which they suggested maybe triggered by insulin resistance. They also showed that maintenance of PGC1*α* levels conferred protection from muscle atrophy by inhibiting transcription of atrophy-related genes, which they noted maybe an indirect effect of a PGC1*α*-mediated increase in mitochondrial content or *β*-oxidative metabolism. Over expression of PGC1*α* in skeletal muscle was shown to preserve mitochondrial function, neuromuscular junctions, and prevent muscle wasting during aging by reducing apoptosis, autophagy, and proteasome degradation [63]. Our data are consistent with these findings, and suggest that the DHA-associated maintenance of PGC1*α* and resulting differences in oxidative metabolism may contribute to improving myotube size and morphology in a high-fat environment.

To determine if markers of oxidative metabolism were maintained similarly to PGC1*α* content by the addition of DHA to palmitate treatment, citrate synthase activity and protein expression of COX-IV were examined as markers of the tricarboxylic acid cycle and electron transport chain, respectively. Contrary to our hypothesis, DHA did not increase citrate synthase activity, either alone, or with palmitate treatment. Conversely, there was a small increase in citrate synthase activity with the palmitate treatment, although most-likely not enough to translate to a physiologically-significant increase in oxidative metabolism. This finding is consistent with previous data demonstrating an increase-in citrate synthase activity in skeletal muscle after high fat feeding [64] and in the muscle of obese animals [65]. Ultimately, however, the current data indicate that DHA did not fully rescue myotube morphology by increasing enzyme activity of the initial step of the tricarboxylic acid cycle. Alternatively, DHA did maintain COX-IV protein levels versus palmitate treatment alone. These data support the idea that maintenance of PGC1*α* also maintains mitochondrial content in the myotubes [62], which would result in preservation of oxidative enzyme protein content instead of necessarily increasing enzyme activity to maintain oxidative capacity and ultimately myotube morphology. Furthermore, Koves and colleagues [66] found that high-fat-induced insulin resistance in animals was associated with decreased expression of PGC1*α* and accumulation of intramuscular acylcarnitines (from *β*-oxidation), while PGC1*α* overexpression in myocytes favored formation of CO_2_ (complete fatty acid oxidation). They suggest that nutrient oversupply leads to an increase in lipid oxidation where the flux of *β*-oxidative by-products overcomes the capacity of the tricarboxylic acid cycle, resulting in incomplete fatty acid oxidation and accumulation of *β*-oxidative intermediates that may contribute to mitochondrial malfunction [66]. Considering these findings, palmitate treatment may trigger a compensatory increase in citrate synthase activity as an attempt to improve complete lipid oxidation in light of increased *β*-oxidative flux without concomitant enhancement of downstream oxidative metabolism (i.e., COX-IV protein abundance) due to decreased PGC1*α* expression. This is further supported by the finding that DHA maintained PGC1*α* and attenuated all of these changes when added to palmitate treatment.

Another aim of this study was to determine if DHA could attenuate the negative effects of palmitate on the insulin signaling pathway in this cell culture model of a high-fat environment. We examined phosphorylation of IRS-1 on serine 636/639, which is inhibitory to the protein, and found that all fatty acid treatments led to 2-3-fold increases in phosphorylation, but without significant differences between treatments or compared to control. Since this measure did not offer much insight into the sensitivity of the insulin signaling pathway, we continued downstream of IRS-1 and measured protein expression and activation of three proteins in the insulin signaling pathway, protein kinase B (Akt), glycogen synthase kinase (GSK) 3*β*, and ribosomal protein S6 (rpS6). Although not statistically significant, Akt phosphorylation and total protein were decreased by ~33% and phospho-GSK3*β* by ~50% with palmitate treatment versus control conditions, while the addition of DHA prevented these decreases. The palmitate-induced decrease in basal and insulin-stimulated Akt activation is consistent with previous research from our lab that demonstrated a ~30% decrease in phospho-Akt^Ser473^/total Akt after treatment of myotubes with 0.75 mM palmitate for 16 hours followed by 10 minutes of serum-stimulation [46]. Sabin et al.[47] reported ~40% decrease in phospho-  Akt^Ser473^ upon insulin stimulation after 24 hours of palmitate treatment but not after treatment with oleate (a monounsaturated fatty acid) in cultured myotubes. This observation highlights the differential effects of unsaturated and saturated fatty acids on the insulin signaling pathway. In addition, others have demonstrated an enhancement of insulin signaling through Akt-mTOR-S6 K-4EBP1 in steers fed with long-chain omega-3 fatty acids [17]. Our finding of increased rpS6 phosphorylation with addition of DHA to the palmitate treatment expands this finding as it is a substrate of S6 K.

## 5. Conclusions

Our data support the idea that the saturated fatty acid palmitate blunted myotube growth, markers of oxidative metabolism, increased intramyocellular lipid content, and caused unresponsiveness to very high concentrations of insulin. However, the omega-3 polyunsaturated fatty acid DHA restored insulin responsiveness, and cellular growth. This was shown in experiments, where the addition of DHA attenuated the palmitate-induced changes in myotube morphology and size, intramyocellular lipid content, and PGC1*α* and COX-IV protein abundance, and these changes were associated with improved basal and insulin-stimulated signaling. Storlien and colleagues first demonstrated a positive effect of fish oil on systemic insulin sensitivity in 1987 [10]. The data in the current study extend these observations and supports a novel hypothesis, that long-chain omega-3 fatty acids may improve insulin signaling in skeletal muscle in a high-fat environment at least in part, by maintaining PGC1*α* protein expression even in the presence of palmitate. However, recent work by Hessvik et al. [59] suggests that mitochondrial mass is independent of fatty acid treatment in myotubes, so the role of mitochondria mass and function on regulation of the beneficial effects in muscle is not yet clear. Future studies are needed to investigate whether omega-3 fatty acids promote oxidative metabolism and preserve mitochondrial mass and quality to prevent insulin resistance and prevent cellular atrophy in a high fat environment.
